# CRISPRi-Library-Guided Target Identification for Engineering Carotenoid Production by *Corynebacterium glutamicum*

**DOI:** 10.3390/microorganisms9040670

**Published:** 2021-03-24

**Authors:** Vanessa L. Göttl, Ina Schmitt, Kristina Braun, Petra Peters-Wendisch, Volker F. Wendisch, Nadja A. Henke

**Affiliations:** Genetics of Prokaryotes, Faculty of Biology & CeBiTec, Bielefeld University, 33615 Bielefeld, Germany; v.goettl@uni-bielefeld.de (V.L.G.); ina.schmitt@uni-bielefeld.de (I.S.); kristina.braun@uni-bielefeld.de (K.B.); petra.peters-wendisch@uni-bielefeld.de (P.P.-W.); n.henke@uni-bielefeld.de (N.A.H.)

**Keywords:** CRISPR interference, carotenoids, CRISPRi, library, metabolic engineering, terpenoids, *Corynebacterium glutamicum*

## Abstract

*Corynebacterium glutamicum* is a prominent production host for various value-added compounds in white biotechnology. Gene repression by dCas9/clustered regularly interspaced short palindromic repeats (CRISPR) interference (CRISPRi) allows for the identification of target genes for metabolic engineering. In this study, a CRISPRi-based library for the repression of 74 genes of *C. glutamicum* was constructed. The chosen genes included genes encoding enzymes of glycolysis, the pentose phosphate pathway, and the tricarboxylic acid cycle, regulatory genes, as well as genes of the methylerythritol phosphate and carotenoid biosynthesis pathways. As expected, CRISPRi-mediated repression of the carotenogenesis repressor gene *crtR* resulted in increased pigmentation and cellular content of the native carotenoid pigment decaprenoxanthin. CRISPRi screening identified 14 genes that affected decaprenoxanthin biosynthesis when repressed. Carotenoid biosynthesis was significantly decreased upon CRISPRi-mediated repression of 11 of these genes, while repression of 3 genes was beneficial for decaprenoxanthin production. Largely, but not in all cases, deletion of selected genes identified in the CRISPRi screen confirmed the pigmentation phenotypes obtained by CRISPRi. Notably, deletion of *pgi* as well as of *gapA* improved decaprenoxanthin levels 43-fold and 9-fold, respectively. The scope of the designed library to identify metabolic engineering targets, transfer of gene repression to stable gene deletion, and limitations of the approach were discussed.

## 1. Introduction

Metabolic engineering offers the possibility to construct production strains that overproduce a valuable compound of interest. Besides the overproduction of native products, also non-native products can be accessed by introducing heterologous pathways. The ability of rational strain engineering holds tremendous value for molecular biology and biotechnology and requires methods to precisely and predictably target genes for expression or repression [1,2].

The clustered regularly interspaced short palindromic repeats (CRISPR) system provides a method for targeted gene editing and gene regulation [3,4]. The CRISPR system from *Streptococcus pyogenes* is applied most often. One approach, called CRISPR interference (CRISPRi) [5], allows for controlled gene repression. It relies on modified protein dCas9 that is catalytically dead due to two amino acid changes in its RuvC and HNH endonuclease domains (D10A and H841A) [5]. The dCas9–sgRNA complex binds to the 20 bp complementary DNA target sequence of the sgRNA, which results in the inhibition of transcription by either blocking transcription elongation or inhibiting transcription initiation [6]. In contrast to a stable gene knockout (deletion), a gene knockdown temporarily suppresses the RNA level of the target gene, and it is a powerful technique to repress genes where a knockout would be lethal [7]. CRISPRi enables fast and robust, but reversible repression of genes. CRISPRi is well suited for functional characterization of essential genes as the knockdown results in reduced, but not abolished activity [7,8,9].

Metabolic engineering targets comprise enzymes that are beneficial for a chosen metabolic trait when their activities are either decreased or increased. The latter can be identified by screening gene overexpression libraries, and the former by screening a collection of deletion mutants or a CRISPRi library for gene repression. Identification of new bottlenecks often requires detailed genetic and biochemical information about the metabolism [10,11]. In this context, the construction of genetic libraries is highly efficient to screen for favorable/unfavorable strain characteristics in a systematic approach. There are genome-wide CRISPRi screenings with 92,000 different sgRNAs in the *Escherichia coli* genome [12,13]. The limiting step of library-based screening is often the readout for the desired phenotype [10], which typically relies on laborious quantifications.

Carotenoids are yellow-to-red-colored natural pigments, and their easy visual readout is suited for phenotypic screening approaches [14,15,16]. Due to their beneficial effects on health and their possible pharmaceutical and nutraceutical applications, carotenoids are also important products for different industries, such as the feed and health industries [17,18]. The global carotenoid market value is expected to reach US$2.0 billion by 2022, with naturally derived carotenoids on the rise.

*Corynebacterium glutamicum* is an excellent platform organism of the bioindustry because of its advantageous traits, such as rapid growth, genetic stability, well-studied genetic background, and the genetic tools for recombinant engineering [19,20]. *C. glutamicum* has already been engineered for the production of a variety of natural and non-natural products from renewable biomass resources, e.g., amino acids [21,22,23]. For the past decades, the industry has relied on the ability of the soil organism *C. glutamicum* to synthesize and secrete amino acids [24]. The central carbon metabolism of *C. glutamicum* has been characterized biochemically and by carbon flux analysis, genetic analysis, and genome-wide studies [25,26]. The metabolic engineering of genes in the central metabolism has been shown to increase fermentative production by *C. glutamicum*, e.g., of l-lysine [27,28,29]. *C. glutamicum* naturally produces the yellow C50 carotenoid decaprenoxanthin and its glucosides [30]. Over the past years, its terpenoid metabolism has been engineered for the production of various isoprenoids, like astaxanthin [31], β-carotene [31], decaprenoxanthin [32], lycopene [33], α-pinene [34], sesquarterpenes [35], patchoulol [36], α-farnesene [37], and (+)-valencene [38]. *C. glutamicum* synthesizes isopentenyl pyrophosphates (isopentenyl diphosphate (IPP) and dimethylallyl diphosphate (DMAPP)) via the 2-methylerythritol 4-phosphate (MEP) pathway [39], and its genome contains a carotenogenic operon encoding the enzymes responsible for terminal decaprenoxanthin biosynthesis starting from the isoprenoid pyrophosphates [40,41]. Metabolic engineering of the central metabolism increases carotenoid production in other host organisms [42,43,44,45], but this has not yet been attempted in *C. glutamicum*.

CRISPR genome editing and CRISPR base editors have been adopted for use in this bacterium [46,47,48,49]. The first CRISPR application to *C. glutamicum* was CRISPRi [50]. CRISPRi was used in several metabolic engineering approaches [7,50,51,52,53,54,55,56], e.g., to improve the production of the amino acids lysine and glutamate [50], as well as butyrate [54] and PHB [52]. The aim of this study was to construct a CRISPRi library for *C. glutamicum* targeting the central metabolism, which should make it useful for the fast identification of targets for rational metabolic engineering to improve the production of a desired compound. The CRISPRi-based library constructed in this study comprised 74 genes, including genes encoding enzymes of glycolysis, the pentose phosphate pathway, and the tricarboxylic acid cycle, regulatory genes, as well as genes of the MEP and carotenoid biosynthesis pathways. As a first application, the CRISPRi library was successfully used to identify targets affecting decaprenoxanthin biosynthesis.

## 2. Materials and Methods

Strains and plasmids used in this study are listed in Table 1. Chemicals were delivered by Carl Roth (Karlsruhe, Germany) if not stated differently. *E. coli* DH5α cells were used for cloning and were cultivated at 37 °C in LB medium. CRISPRi library experiments were carried out in the prophage-cured MB001 strain [57]. Experiments adapting the results of the library were carried out in the wild-type ATCC 13032. Precultures of *C. glutamicum* strains were grown in the brain heart infusion (BHI) complex medium (37 g L^−1^) supplemented with 10 g L^−1^ glucose overnight. Main cultures for CRISPRi library screening were grown in CGXII minimal medium [58] supplemented with 40 g L^−1^ of glucose supplemented with 1 mM IPTG and 0.25 µg mL^−1^ of anhydrotetracycline (aTc) for induction after washing in minimal medium. Cultures were inoculated to an initial OD_600nm_ of 1 using a Shimadzu UV-1202 spectrophotometer (Duisburg, Germany). Cultivations were performed in 1 mL in the Biolector^®^flowerplate microcultivation system (m2p-labs GmbH, Baesweiler, Germany) at 1100 rpm and 30 °C. *C. glutamicum* WT and *C. glutamicum* WT Δ*sdhCAB* were cultivated in 50 mL of CGXII plus 40 g L^−1^ of glucose in baffled shake flasks. *C. glutamicum* WT and *C. glutamicum* WT ∆*aceE* were cultivated in 50 mL of CGXII plus 40 g L^−1^ of glucose and 20 g L^−1^ of potassium acetate in baffled shake flasks. *C. glutamicum* WT and strains WT Δ*pgi,* WT Δ*gapA*, and WT Δ*sugR* were cultivated in 1 mL of CGXII plus 40 g L^−1^ of glucose in Duetz plates at 30 °C and 220 rpm. As an antibiotic, chloramphenicol (VWR, Darmstadt, Germany) was added to the CRISPRi plasmids in concentrations of 7.5 μg mL^−1^ for *C. glutamicum* cultures and 30 μg mL^−1^ for *E. coli* cultures.

### 2.1. Construction of the CRISPRi Vector System

For construction of the CRISPRi library plasmid pS_dCas9, the vector pRG_dCas9 [46] was restricted with PstI and SalI (NEB, Frankfurt, Germany) and dephosphorylated (Antarctic phosphatase, New England Biolabs, Frankfurt, Germany). The sequences of the dCas9 handle and terminator from *S. pyogenes* were amplified by high-fidelity PCR (All-in HiFi, Kraichtal, Germany) from the plasmid piCas [66] with the oligonucleotides vgag and vgam (Table S1), and the PCR amplicon was purified with a PCR and gel extraction kit (Macherey-Nagel, Düren, Germany). The PCR product was cloned into the PstI- and SalI-restricted, dephosphorylated vector pRG_dCas9 by Gibson Assembly [67], resulting in plasmid pS_dCas9. Standard genetic procedures were performed as described previously [68].

### 2.2. Construction of the CRISPRi Library

The sgRNAs were designed to contain a 20 bp region homologous to the non-template strand of the chosen DNA targets. The genome sequence of *C. glutamicum* ATCC 13,032 [39] was used as a basis for the selection of the 20 bp targeting sequences using the CRISPy-web tool [69]. CRISPRi library plasmids were constructed in *E. coli* DH5α through the annealing oligo method, with single-stranded oligonucleotides covering sgRNA (20 bp) and 20 bp overlaps with plasmid pS_dCas9. Equal volumes (5 µL) of equimolar oligonucleotides (100 µM) were mixed with the annealing buffer (990 µL), and the mixture was incubated at 95 °C for 5 min and then cooled down to room temperature. The CRISPRi library plasmid pS_dCas9 was restricted with PstI (NEB, Frankfurt, Germany) and dephosphorylated (Antarctic phosphatase, New England Biolabs, Frankfurt, Germany) before the double-stranded oligonucleotides were annealed by the Gibson Assembly method [67]. The concentration of DNA was measured with an ND-1000 spectrophotometer (Thermo Fisher Scientific, Schwerte, Germany). The oligonucleotides (Table S1) used in this study were obtained from Metabion (Planegg/Steinkirchen, Germany). *E. coli* DH5α cells were transformed by heat shock after preparation of CaCl_2_-competent cells [70]. Transformants were screened by colony PCR, and plasmids were isolated by a plasmid miniprep kit (GeneJET, Thermo Fisher Scientific, Schwerte, Germany). Library vectors were confirmed by sequencing with oligonucleotides vgai and vgaj (Table S1). *C. glutamicum* cells were transformed by electroporation [71].

### 2.3. Construction of C. glutamicum Deletion Mutants

For deletion of the *sdhCAB* operon, the suicide vector pK19*mobsacB* was used [65]. The genomic flanking regions of *sdhCAB* were amplified from the genomic DNA of *C. glutamicum* WT using the oligonucleotide pairs del-sdhCAB1/del-sdhCAB2 and del-sdhCAB3/del-sdhCAB4 (Table S1). The PCR amplicons were purified, linked by crossover PCR, and subsequently cloned into a *SmaI*-restricted pK19*mobsacB*. The resulting deletion vector pK19*mobsacB*-*sdhCAB* was introduced into *C. glutamicum* via trans conjugation with *E. coli* S17-1 [65]. Deletion of *sdhCAB* was achieved by two-step homologous recombination using the respective deletion vector, as previously described [70]. Integration of the vector into one of the gene-flanking regions represents the first recombination event and was selected via kanamycin resistance. Integration of the vector into the genome results in sucrose sensitivity due to levansucrase, encoded by *sacB*. Selection for the second recombination event, loss of the vector, was carried out via sucrose resistance. Deletion of *sdhCAB* was verified via sequencing with primers del-sdhCAB-5 and del-sdhCAB-6 (Table S1).

### 2.4. Quantification of the mRNA Levels of Targeted Cells by CRISPRi

RNA levels were determined by quantitative reverse transcription PCR (qRT-PCR). Total RNA was isolated from *C. glutamicum* strains growing exponentially in CGXII medium. Biological triplicates were analyzed. Aliquots of 500 µL were centrifuged at 14,000 rpm for 15 s (Eppendorf centrifuge 5810 R), and the pellets were immediately frozen in liquid nitrogen and stored at −80 °C until further use. For RNA isolation, the samples were homogenized by resuspending the cells in 100 μL of TE buffer (10 mM Tris-HCl, 1 mM EDTA; pH 8) containing 5 mg mL^−1^ of lysozyme. After incubation at 37 °C for 30 min, total RNA was extracted using a NucleoSpin^®^ RNA kit (Macherey-Nagel, Düren, Germany) according to the manufacturer’s instructions. After extraction, RNA samples were treated with DNase restriction using RNase-free DNase Set and RNeasy MinElute kits (Qiagen, Hilden, Germany) to eliminate possible genomic DNA contamination. The total RNA concentration was measured using a spectrophotometer (NanoDrop^®^, ND-1000; ThermoFisher Scientific, Schwerte, Germany). Quality control was performed to determine the purity and integrity of isolated RNA.

Equal amounts of 50 ng of each sample were used to perform cDNA synthesis. qRT-PCR was performed using the SensiFAST^TM^ SYBR^®^ No-ROX One-Step Kit (Bioline, London, UK) and the CFX96 cycler system (Bio-Rad, Hercules, CA, USA). The temperature profile was (1) 45 °C for 10 min; (2) 95 °C for 2 min; (3) 40 cycles of 95 °C for 5 s, 56 °C for 15 s, and 72 °C for 15 s; and (4) melt curve analysis with measures between 65 °C and 95 °C. The used primers for qRT-PCR are listed in Table S1. The ΔCq method was used in calculations [72,73].

### 2.5. Carotenoid Quantification

Carotenoid production was analyzed by high-performance liquid chromatography (HPLC) analysis. Carotenoids were extracted from the cell fraction using a methanol:acetone (7:3) mixture. Extraction was performed at 60 °C and 600 rpm for 30 min. After centrifugation at 14,000 rpm and 10 min, the supernatant was used for HPLC analysis. The Agilent 1200 series system (Agilent Technologies, Waldbronn, Germany) was used with a reversed-phase precolumn (LiChrospher 100 RP18 EC-5, 40 × 4 mm) (CS-Chromatographie, Langerwehe, Germany) and a reversed-phase main column (LiChrospher 100 RP18 EC-5, 125 × 4 mm) (CS-Chromatographie, Langerwehe, Germany), and methanol (A) and methanol:water (9:1) (B) were used as mobile phases. Carotenoids were detected with a diode array detector (DAD) by recording of the UV–visible (Vis) spectrum. A gradient at a flow rate of 1.5 mL min^−1^ was used as follows: 0 min B: 0%, 10 min B: 100%, and 32.5 min B: 100%. The decaprenoxanthin measured and presented in the Results section (Section 3) is di-glycosylated decaprenoxanthin. For quantification, the extracted wavelength chromatogram at λ_max_ of 470 nm was used and standardized with β-carotene (Sigma-Aldrich, Steinheim, Germany).

## 3. Results

### 3.1. Design and Initial Testing of a CRISPRi Library for Gene Repression in C. glutamicum

This work aimed to design, construct, and test a CRISPRi library suitable for gene repression screening in *C. glutamicum*. In principle, the approach is generalizable, but here, we focused on scoring the effect of repressing genes of the central carbon metabolism and carotenogenesis as well as regulatory genes on the biosynthesis of decaprenoxanthin, the natural pigment of *C. glutamicum*.

#### 3.1.1. Construction of a Vector CRISPRi Library for *C. glutamicum*

**Figure 1:**
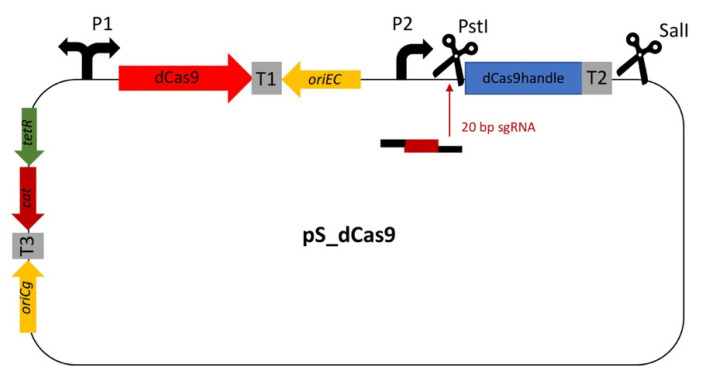
Construction of the dual-inducible CRISPRi expression plasmid pS_dCas9. Adapted pRG_dCas9 plasmid carrying the dCas9 handle followed by the terminator from *S. pyogenes* amplified from plasmid piCas between the PstI and SalI restriction sites. A 20 bp sgRNA sequence can be inserted in the PstI cloning site. For multiplexing of more than one specific sgRNA, the restriction site SalI after the first sgRNA-cs can be used. It has chloramphenicol resistance. P1: *tetR/tetA* promotor; P2: *tac* promotor; T1: rrnB T1 terminator; T2: terminator from *S. pyogenes*; T3: lambda terminator; ori*Ec*: p15A; ori*Cg*: pCG1; cat: chloramphenicol resistance.

The CRISPRi library was based on pRG_dCas9 [56]. To ease library preparation, the dCas9 handle followed by the terminator from *S. pyogenes* was amplified from plasmid piCas [66] and inserted between the PstI and SalI restriction sites, resulting in vector pS_dCas9 (Figure 1). The various gene-specific 20 bp sgRNA sequences of the target library (Table S2) were generated from oligonucleotides by the annealing oligo method before being inserted into the PstI cloning site.

#### 3.1.2. Testing of the CRISPRi Library Vector for the Repression of *crtR*

**Figure 2:**
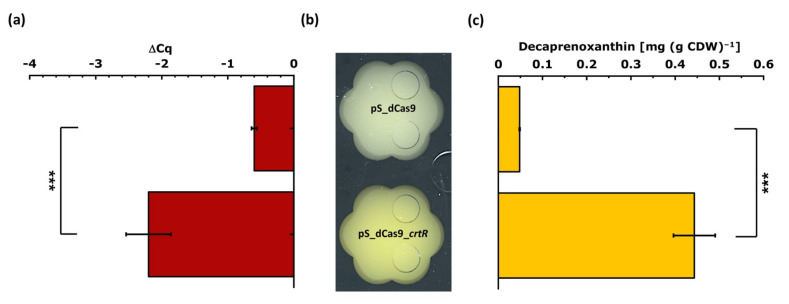
Testing the CRISPRi system for identification of metabolic engineering targets relevant for carotenoid production. Strain *C. glutamicum* MB001(pS_dCas9_*crtR*) for CRISPRi-mediated repression of *crtR* was compared to the empty vector carrying control strain MB001(pS_dCas9) with respect to (**a**) *crtR* RNA levels, (**b**) color phenotypes, and (**c**) cellular decaprenoxanthin content. For qRT-PCR analysis, exponentially growing cells were harvested and *sigA* was used as a reference (**a**). The color phenotype (**b**) was judged by visual inspection after growth in the Biolector^®^flowerplate microcultivation system. Cells were grown in 40 g L^−1^ of glucose CGXII minimal medium for 28 h and induced at 0 h with 1 mM IPTG and 0.25 µg mL^−1^ of aTc. The cellular decaprenoxanthin content (**c**) is given as ß-carotene equivalents, as determined by HPLC analysis. Mean values and standard deviations of three biological replicates are given. The *p*-value of <0.001 (***) was calculated using Student’s *t*-test (two sided, unpaired).

To test the sensitivity and function of the CRISPRi library vector, the repression of *crtR* coding for the transcriptional repressor CrtR of the carotenogenic *crt* operon [63] was chosen. We have shown previously that deletion of *crtR* in *C. glutamicum* MB001 increased the accumulation of the native carotenoid pigment decaprenoxanthin about 30-fold [63]. Therefore, we assumed that targeting *crtR* by CRISPRi will increase decaprenoxanthin biosynthesis. The sgRNA sequence for *crtR* (and later for all tested genes) was chosen according to the following strategy: the sgRNA (i) was identified using CRISPy-web [69] targeting the non-template strand, (ii) is located in the coding sequence of the target gene for inhibition of transcription elongation, (iii) preferably is in the 5′ proximal region of the coding sequence, and (iv) is unique in the *C. glutamicum* genome. The plasmid pS_dCas9_*crtR* and the empty vector pS_dCas9 were used to transform *C. glutamicum* MB001, and the resulting recombinant strains were cultivated in glucose minimal medium with 1 mM IPTG for induction of the sgRNA and with 0.25 µg mL^−1^ of aTc for induction of the dCas9 gene. Exponentially growing cells were harvested for RNA extraction, while the decaprenoxanthin content was quantified after cultivation for 28 h. After qRT-PCR, the ΔCq value was calculated using the vegetative RNA polymerase sigma factor gene *sigA* as a reference. The qRT-PCR analysis revealed a statistically significant and about fourfold lower *crtR* RNA level upon targeting *crtR* using pS_dCas9_*crtR* (Figure 2a).

Phenotypically, *C. glutamicum* MB001(pS_dCas9_*crtR*) indeed showed a more intense yellow pigmentation than strain MB001(pS_dCas9) (Figure 2b). Accordingly, HPLC analysis revealed an about ninefold, significantly (*p*-value < 0.001) higher decaprenoxanthin content (0.44 ± 0.05 mg (g CDW)^−1^) for *C. glutamicum* MB001(pS_dCas9_*crtR*) as compared to the empty vector carrying the control strain MB001(pS_dCas9) (0.05 ± 0.00 mg (g CDW)^−1^; Figure 2c). Thus, based on visual observation of pigmentation, qRT-PCR analysis of *crtR* RNA levels, and decaprenoxanthin quantification, the CRISPRi system proved suitable to score the effect that repression of a gene of interest has on carotenogenesis. To test the duration of the inhibitory effect of the CRISPRi targeting *crtR*, serial transfers from a culture grown for 6 generations in the presence of inducers to a medium without inducers were performed. CRISPRi targeting of *crtR* visibly increased decaprenoxanthin levels to above those of the empty vector control strain in both serial cultures. However, the effect faded gradually from serial transfer one (5 generations without inducers) to serial transfer two (8 generations without inducers) (Figure S1).

### 3.2. Characterization of a CRISPRi Library to Interrogate 74 Target Genes with Potential Relevance for Carotenogenesis in C. glutamicum

**Figure 3:**
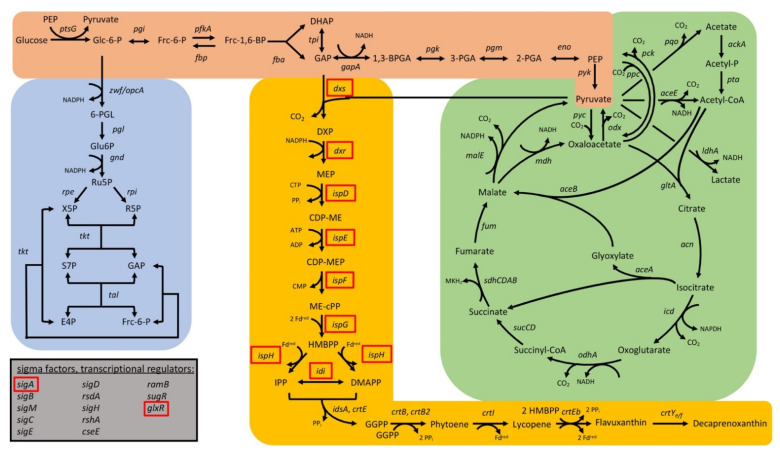
Scheme of the central carbon metabolism and carotenogenesis in *C. glutamicum* with glycolysis (orange shading), the pentose phosphate pathway (blue shading), the TCA cycle (green shading), as well as the MEP pathway and the carotenogenesis (yellow shading). Gene names are given next to the reactions catalyzed by their gene products. The corresponding gene identifiers can be found in Table 1. Essential genes are depicted with a red box. *aceA*: isocitrate lyase; *aceB*: malate synthase; *aceE*: pyruvate dehydrogenase E1 component; *ackA*: acetate kinase; *acn*: aconitase; *crtB*: phytoene synthase; *crtB2*: phytoene synthase 2; *crtE*: geranylgeranyl-diphosphate synthase; *crtEb*: lycopene elongase; *crtI*: phytoene desaturase; *crtI2*: phytoene desaturase; *crtY_e/f_*: C50 carotenoid epsilon cyclase; *cseE*: anti-sigma factor E; *dxr*: 1-deoxy-D-xylulose 5-phosphate reductoisomerase; *dxs*: 1-deoxyxylulose-5-phosphate synthase; *eno*: enolase; *fba*: fructose-1,6-bisphosphate aldolase; *fbp*: fructose 1,6-bisphosphatase; *fum*: fumarase; *gapA*: glyceraldehyde-3-phosphate dehydrogenase A; *gltA*: citrate synthase; *glxR*: global transcriptional regulator; *gnd*: 6-phosphogluconate dehydrogenase; *icd*: isocitrate dehydrogenase; *idi*: isopentenyldiphosphate isomerase; *idsA*: geranylgeranyl diphosphate synthase; *ispD*: 2-C-methyl-D-erythritol 4-phosphate cytidylyltransferase; *ispE*: 4-diphosphocytidyl-2-C-methyl-D-erythritol kinase; *ispF*: 2-C-methyl-D-erythritol 4-phosphate cytidylyltransferase; *ispG*: 4-hydroxy-3-methylbut-2-en-1-yl diphosphate synthase; *ispH*: 4-hydroxy-3-methylbut-2-enyl diphosphate reductase; *ldh*: NAD-dependent L-lactate dehydrogenase; *malE*: malic enzyme; *mdh*: malate dehydrogenase; *odhA*: oxoglutarate dehydrogenase subunit A; *odx*: oxaloacetate decarboxylase; *opcA*: glucose-6-phosphate dehydrogenase; *pck*: phosphoenolpyruvate carboxykinase; *pfkA*: 6-phosphofructokinase; *pgi*: glucose-6-phosphate isomerase; *pgk*: phosphoglycerate kinase; *pgl*: 6-phosphogluconolactonase; *pgm*: phosphoglucomutase; *ppc*: phosphoenolpyruvate carboxylase; *pqo*: pyruvate quinone oxidoreductase; *pta*: phosphotransacetylase; *ptsG*: glucose-specific enzyme II BC component of PTS; *pyc*: pyruvate carboxylase; *pyk*: pyruvate kinase; *ramB*: transcriptional regulator of acetate metabolism A; *rpe*: ribulose-5-phosphate epimerase; *rpi*: phosphopentose isomerase; *rsdA*: anti-sigma factor D; *rshA*: anti-sigma factor H; *sdhABDC*: succinate dehydrogenase subunits A, B, C, and D; *sigA*: sigma factor A; *sigB*: sigma factor B; *sigC*: sigma factor C; *sigD*: sigma factor D; *sigE*: sigma factor E; *sigH*: sigma factor H; *sigM*: sigma factor M; *sucCD*: succinyl-CoA synthetase beta and alpha subunits; *sugR*: transcriptional regulators of sugar metabolism; *tal*: transaldolase; *tkt*: transketolase; *tpi*: triosephosphate isomerase; *zwf*: glucose-6-phosphate 1-dehydrogenase. 1,3-BPGA: 1,3-bisphosphate glycerate; 2-PGA:2-phosphate glycerate; 3-PGA: 3-phosphate glycerate; 6-PGI: 6-phosphogluconolactone; acetyl-P: acetyl-phosphate; CDP-ME: 4-diphosphocytidyl-2-methylerythritol; CDP-MEP: 4-diphosphocytidyl-2-methylerythritol 2-phosphate; DHAP: dihydroxyacetone phosphate; DMAPP: dimethylallyl diphosphate; DXP: 1-deoxy-D-xylulose 5-phosphate synthase; E4P: erythrose-4-phosphate; Frc-1,6-BP: fructose-1,6-bisphosphate; Frc-6-P: fructose-6-phosphate; GAP: glyceraldehyde 3-phosphate; GGPP: geranylgeranyl diphosphate; Glc-6-P: glucose-6-phosphate; Glu6P: 6-phosphogluconate; HMBPP: (*E*)-4-hydroxy-3-methyl-but-2-enyl diphosphate; IPP: isopentenyl diphosphate; ME-cPP: 2-methylerythritol 2,4-cyclodiphosphate; MEP: 2-methylerythritol 4-phosphate; PEP: phosphoenolpyruvate; R5P: ribose-5-phosphate; Ru5P: ribulose-5-phosphate; S7P: sedoheptulose 7-phosphate;X5P: xylulose-5-phosphate.

**Figure 4:**
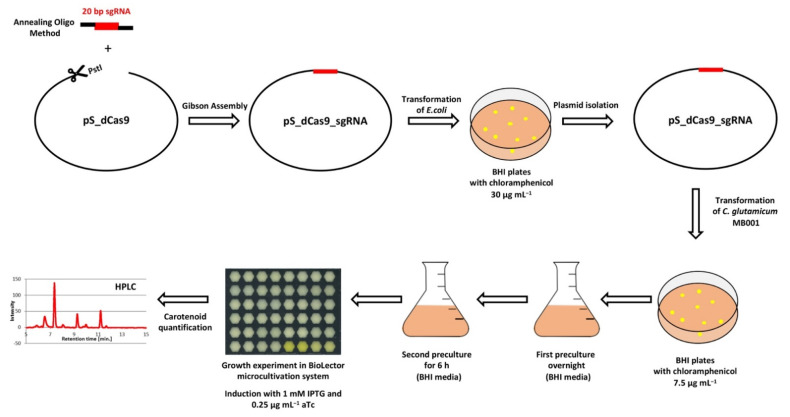
Workflow of CRISPRi library construction and screening in *C. glutamicum* MB001.

In total, 74 target genes from *C. glutamicum* were repressed by CRISPRi; the growth parameters of the respective strains are listed in Table S3. The CRISPRi library comprising a subset of genes from central carbon metabolism as well as regulatory genes was selected to cover glycolysis (19 gene targets), the pentose phosphate pathway (8 gene targets), and the tricarboxylic acid (TCA) cycle (17 gene targets) for general use in metabolic engineering (Figure 3). With the goal to improve isoprenoid and carotenoid production as the chosen application example in this study, genes were selected to cover the MEP pathway of isoprenoid pyrophosphate biosynthesis (6 genes targets) as well as terminal carotenogenesis (8 gene targets) (Figure 3). The CRISPRi library approach is commensurate with multivariate modular metabolic engineering [74]. The workflow of the approach is illustrated in Figure 4.

#### 3.2.1. CRISPRi-Based Repression of Genes of the MEP Pathway and of Carotenogenesis-Reduced Decaprenoxanthin Pigmentation

**Figure 5:**
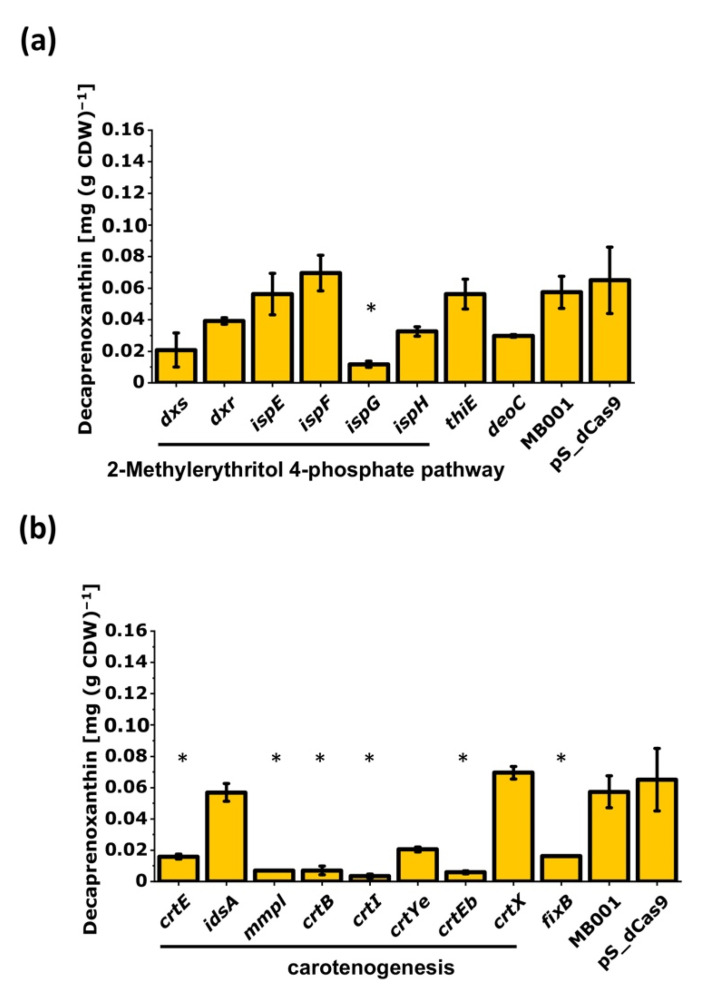
Influence of CRISPRi-mediated repression of genes of the MEP pathway (**a**) or of carotenogenesis (**b**) on decaprenoxanthin production by *C. glutamicum*. In addition, *thiE*, *deoC*, and *fixB* were analyzed. Mean values of biological duplicates are given. Statistical analysis was calculated with ANOVA against all measured decaprenoxanthin production of *C. glutamicum* MB001 from all Biolector^®^flowerplates and is marked by a star (*). As a reference, the decaprenoxanthin production of the empty vector strain *C. glutamicum* MB001 (pS_dCas9) in biological duplicates of the corresponding experiment is shown. For abbreviations, see Figure 3.

Repression of genes of the MEP pathway and of carotenogenesis was expected to reduce pigmentation. To test this hypothesis, the respective CRISPRi library transformants were analyzed for decaprenoxanthin production. Indeed, repression of *ispG* lowered the cellular decaprenoxanthin content significantly, whereas repression of the other MEP pathway genes did not affect decaprenoxanthin content in a statistically significant manner, albeit some reduction was observed (Figure 5a). CRISPRi targeting of the *crt* operon genes *crtE*, *mmpL*, *crtB*, *crtI*, and *crtEb* reduced decaprenoxanthin biosynthesis significantly (Figure 5b). This was observed neither upon targeting *idsA* nor upon targeting *crtX.* These genes are not part of the *crt* operon. While CRISPRi repression of *crtX* did not reduce the decaprenoxanthin level (Figure 5b), unglucosylated instead of diglucosylated decaprenoxanthin accumulated, which was not observed for all other strains. This finding was commensurate with CrtX functioning as decaprenoxanthin glucosyltransferase [33].

**Figure 6:**
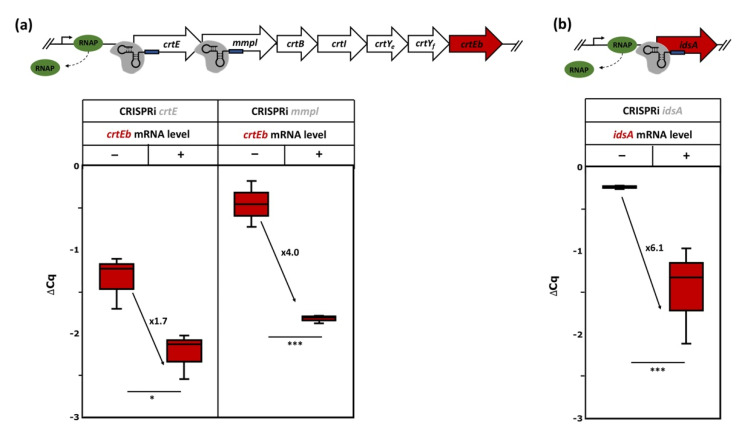
Schematic representation of the *crt* operon and *idsA* and qRT-PCR analysis of *crtEb* RNA levels upon CRISPRi targeting of *crtE* or *mmpL* (**a**) or of *idsA* RNA levels upon CRISPRi targeting of *idsA* (**b**). Cells exponentially growing in 40 g L^−1^ of glucose CGXII minimal medium with (+) or without (−) induction using 1 mM IPTG and 0.25 µg mL^−1^ of aTc were analyzed. Mean values and standard deviations of three biological replicates are given. The *p*-values of <0.001 (***), and <0.05 (*) were calculated using Student’s *t*-test (two sided, unpaired). For abbreviations, see Figure 3.

In the case of *idsA*, qRT-PCR analysis revealed that repression reduced the mRNA level by about sixfold (Figure 6), while decaprenoxanthin biosynthesis was hardly affected. Possibly, CrtE compensates for the loss of IdsA, since *crtE* and *idsA* both encode geranylgeranyl diphosphate (GGPP) synthases [75]. The reduced decaprenoxanthin level observed upon CRISPRi repression of *crtE* may either be due to IdsA not being able to compensate for reduced CrtE levels or be due to polar effects on downstream *crt* operon genes, since *crtE* is the first gene in the *crt* operon. To directly test for downstream effects, qRT-PCR analysis of the RNA level of *crtEb*, the last gene of the *crtE*-*mmpL*-*crtB*-*crtI*-*crtY_e_*-*crtY_f_*-*crtEb* operon, was performed in strains where CRISPRi repressed either the first or the second *crt* operon gene (*crtE* or *mmpL*). Indeed, the *crtEb* mRNA level was significantly reduced upon CRISPRi targeting of *crtE* as well as *mmpL* (Figure 6); thus, the observed reduced decaprenoxanthin biosynthesis upon CRISPRi repression of *crtE* and *mmpL* is, at least in part, not due to lowered CrtE or MmpL levels but due to repression of the whole *crt* operon.

Three further genes were analyzed. Since the MEP pathway provides precursors also for thiamin biosynthesis, repression of the first gene of the thiamin biosynthesis operon *thiEOSGF* coding for thiamine-phosphate pyrophosphorylase ThiE was studied. The gene for deoxyribose-phosphate aldolase (*deoC*) that catalyzes a condensation reaction similar to that of the MEP pathway enzyme Dxs was also chosen. Finally, *fixB* coding for a protein of the electron transfer flavoprotein (ETF) family with some resemblance to a novel β-cyclase enzyme CruA from *Chlorobium tepidum* [76] was repressed. Upon CRISPRi targeting of *thiE*, *deoC*, and *fixB,* decaprenoxanthin levels were unaffected, somewhat reduced, and significantly decreased, respectively (Figure 5).

#### 3.2.2. CRISPRi-Based Repression of Genes of the Central-Carbon-Metabolism-Identified Supply of GAP and Entry into the Pentose Phosphate Pathway as Potential Bottlenecks in Decaprenoxanthin Biosynthesis

**Figure 7:**
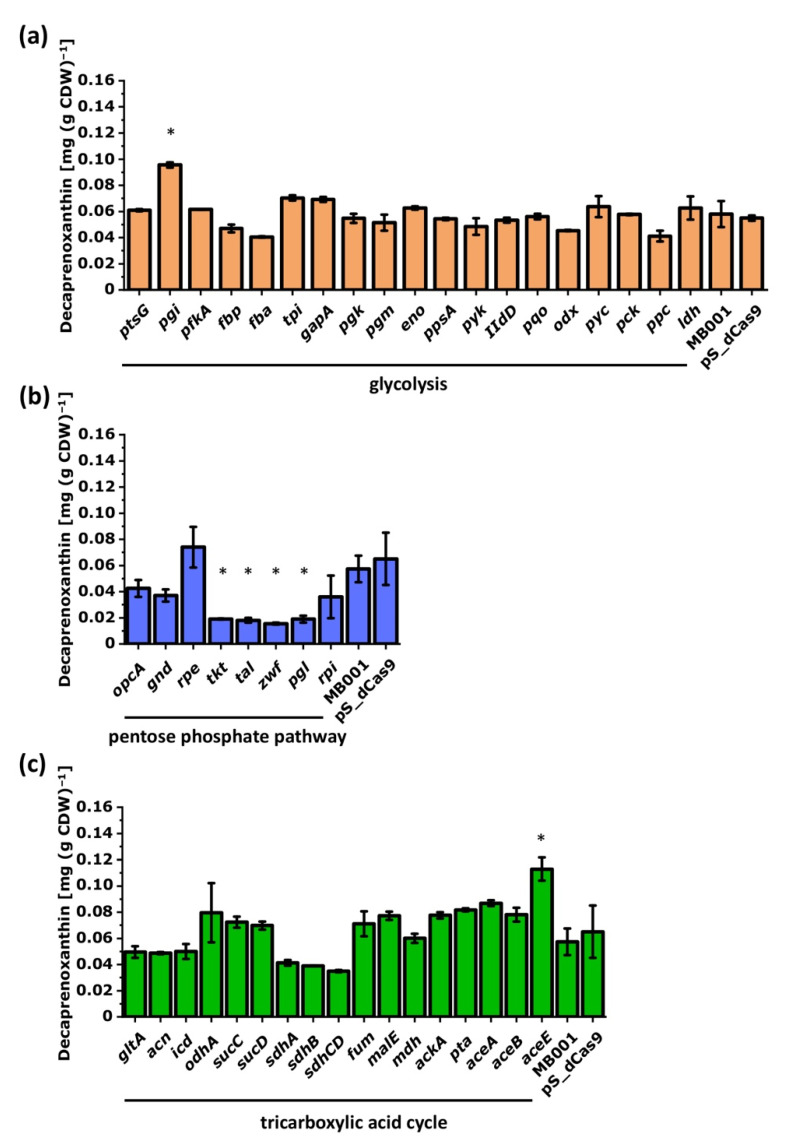
Influence of CRISPRi-mediated repression of genes of glycolysis (**a**), the pentose phosphate pathway (**b**), or the TCA cycle and glyoxylate shunt (**c**) on decaprenoxanthin biosynthesis. Mean values of biological duplicates are given. Statistical analysis was calculated with ANOVA against all measured decaprenoxanthin production of *C. glutamicum* MB001 from all Biolector^®^flowerplates and is marked by a star (*). As a reference, the decaprenoxanthin production of the empty vector strain *C. glutamicum* MB001 (pS_dCas9) in biological duplicates of the corresponding experiment is shown. For abbreviations, see Figure 3.

To identify the influence of the central metabolism on the decaprenoxanthin production, selected genes were repressed by CRISPRi and decaprenoxanthin was quantified (Figure 7). While the repression of the glycolytic gene *pgi* improved decaprenoxanthin production significantly, CRISPRi targeting of the pentose phosphate pathway genes *tkt*, *tal*, *zwf*, and *pgl* negatively affected decaprenoxanthin pigmentation (Figure 7b). Repression of *aceE* encoding a subunit of the pyruvate dehydrogenase complex that is relevant for carbon entry into the TCA cycle improved decaprenoxanthin biosynthesis (Figure 7c). CRISPRi targeting of the TCA cycle genes *sdhA*, *sdhB,* and *sdhCD* lowered decaprenoxanthin accumulation (Figure 7c) in each case; however, these changes were not statistically significant.

#### 3.2.3. Interrogation of Regulatory Genes by CRISPRi with Respect to Carotenoid Biosynthesis in *C. glutamicum*

**Figure 8:**
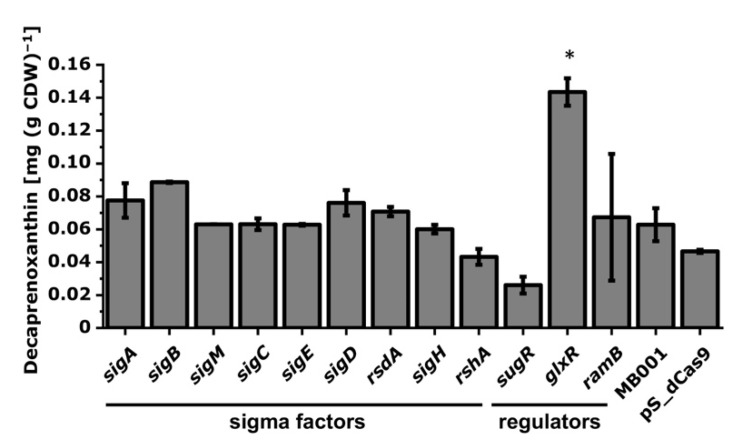
Influence of CRISPRi-mediated repression of RNA polymerase sigma factor and transcriptional regulator genes on decaprenoxanthin biosynthesis. Mean values of biological duplicates are given. Statistical analysis was calculated with ANOVA against all measured decaprenoxanthin production of *C. glutamicum* MB001 from all Biolector^®^flowerplates and is marked by a star (*). As a reference, the decaprenoxanthin production of the empty vector strain *C. glutamicum* MB001 (pS_dCas9) in biological duplicates of the corresponding experiment is shown. For abbreviations, see Figure 3.

*C. glutamicum* possesses seven RNA polymerase sigma factors, which in part are regulated by their cognate anti-sigma factors [39]. The deletion of the sigma factor gene *sigB* is known to increase carotenoid production [77]. To identify the influence of regulatory genes on carotenoid production in *C. glutamicum*, RNA polymerase sigma factor and anti-sigma factor genes and the genes for the transcriptional regulators of carbon metabolism SugR, GlxR, and RamB were chosen. CRISPRi targeting of *sigB* and *sugR* increased and decreased, respectively, the cellular decaprenoxanthin content; however, the effects were not statistically relevant (Figure 8). Notably, CRISPRi-mediated repression of *glxR* significantly increased the cellular decaprenoxanthin by about twofold (Figure 8).

### 3.3. Deletion of Selected Target Genes Identified by CRISPRi Repression

**Figure 9:**
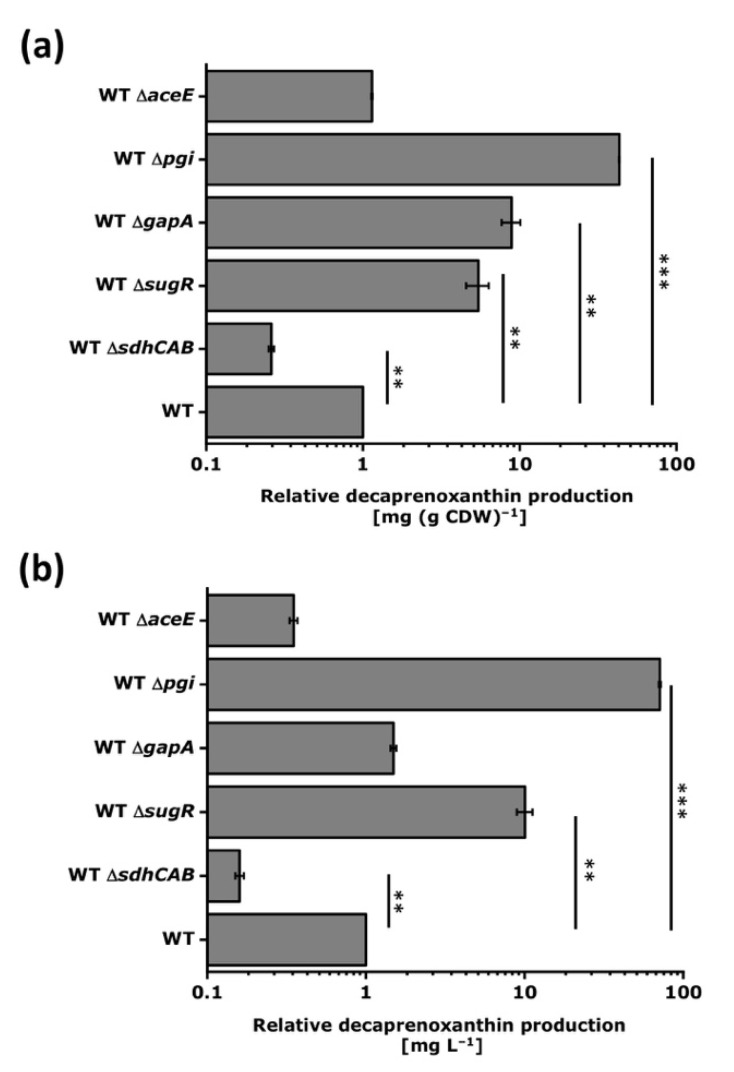
Relative decaprenoxanthin production by *C. glutamicum* strains carrying various deletions. Cells were grown in 40 g L^−1^ of glucose CGXII minimal medium. *C. glutamicum* WT Δ*aceE* was grown with 20 g L^−1^ of potassium acetate and 40 g L^−1^ of glucose in CGXII minimal medium. Decaprenoxanthin production was determined by HPLC analysis. Due to the medium differences, decaprenoxanthin contents in mg g^−1^ CDW (**a**) and concentrations in mg L^−1^ (**b**) were normalized to the values obtained with the parental WT strain. Mean values and standard deviations of triplicates are given. The *p*-values of <0.001 (***), and <0.01 (**) were calculated using Student’s t-test (two sided, unpaired). For abbreviations, see Figure 3.

Gene deletion is a favored metabolic engineering strategy since the constructed strains are genetically stable (except when suppressor mutations occur in *trans*). Gene deletion results in the complete loss of function (knockout), while CRISPRi-mediated repression reduces gene function (knockdown). A gene can be repressed by CRISPRi, but not deleted, if its function is essential. The MEP pathway genes, the RNA polymerase sigma factor A gene *sigA*, and, according to some reports, the regulatory gene *glxR* cannot be deleted in *C. glutamicum*. Deletion of the *crt* operon is known to abolish decaprenoxanthin biosynthesis [33,40], thus supporting the evidence obtained by CRISPRi (Figure 5). Deletion of *crtX* is known to result in accumulation of unglucosylated decaprenoxanthin [32]. In addition, we chose other genes to study the effect of gene deletion on decaprenoxanthin production, i.e., *pgi*, *gapA*, and *aceE*, as their deletion was expected to increase decaprenoxanthin levels, as well as *sdhABCD* and *sugR*, since their deletion was expected to reduce carotenoid biosynthesis (Figure 9).

In line with the CRISPRi results, deletion of the *sdhABCD* operon significantly reduced decaprenoxanthin levels in comparison to *C. glutamicum* WT, while deletion of *pgi* and *gapA* improved the cellular decaprenoxanthin content significantly (Figure 9a). Since the *gapA* deletion mutant showed impaired growth, the decaprenoxanthin concentration in the culture was not increased (Figure 9b). Therefore, the pleiotropic effects of *gapA* deletion nullified the increased decaprenoxanthin content per cell biomass as the biomass concentration was reduced. SugR represses many target genes and binds their promoter DNA regions with different affinities in vitro [78]; thus, besides pleiotropic effects, gradual regulatory differences may be expected as well. In contrast to the CRISPRi results, deletion of *sugR* increased the decaprenoxanthin content in comparison to the control strain fivefold. Although not understood mechanistically, these results indicate that the complete absence of SugR upon gene deletion is beneficial, while SugR levels below those of the WT strain upon CRISPRi targeting may limit decaprenoxanthin biosynthesis.

Deletion of *aceE* is known to be possible, but the resulting mutant requires a source of acetyl-CoA, such as acetate, for growth [60]. Therefore, with glucose minimal medium, the positive effect observed of CRISPRi targeting *aceE* could not be tested by the deletion of *aceE* since *aceE* deletion is conditionally lethal under these conditions. To circumvent this problem, the *aceE* mutant was assayed for decaprenoxanthin biosynthesis in glucose medium supplemented with acetate. The decaprenoxanthin content per biomass of the *aceE* mutant grown with glucose and acetate was, however, not higher than that of the WT strain grown with glucose.

Taken together, these results highlight the advantage of CRISPRi screening over deletion analysis. On the one hand, it allows for fast target gene identification for metabolic engineering, and transfer to the construction of genetically stable strains by gene deletion often is straightforward (*sdhCAB*, *pgi*, *gapA*). On the other hand, CRISPRi is a well-suited method to analyze essential genes, conditionally lethal genes such as *aceE*, as well as pleiotropic genes or regulatory genes.

## 4. Discussion

In this study, the first *C. glutamicum* CRISPRi library for the repression of 74 genes (e.g., those for central metabolism, coding for global regulators and RNA polymerase sigma factors, besides the genes for carotenoid biosynthesis) was constructed and tested to identify targets affecting carotenogenesis. As expected, decaprenoxanthin levels were reduced when the MEP pathway or carotenogenesis genes were repressed. In addition, eight new targets to improve carotenoid production were identified. Using deletions instead of CRISPRi repression for five selected genes supported CRISPRi results for some genes (*sdhABCD*, *pgi*, *gapA*). This was not the case for the conditionally lethal *aceE* deletion and for the pleiotropic deletion of the global regulatory gene *sugR*.

CRISPRi screenings were useful to identify promising targets for increased carotenoid production in other organisms [79,80]. Clearly, CRISPRi repression is complementary to gene deletion, not only because CRISPRi allows functional analysis of essential genes by assigning phenotypes as a consequence of their repression, but also because regulatory genes can be titrated, resulting in phenotypes that are intermediate between the levels in the wild type and the null levels in a deletion mutant. In *C. glutamicum* systems biology, loss of function analysis by gene deletions is often coupled with transcriptome, proteome, metabolome, and fluxome studies to gain insight, for example, into regulons and modulons [81]. Similarly, CRISPRi libraries may contribute to a systems-level understanding of an organism when coupled with omics experiments, allowing one to assess responses to gradual rather than absence/presence perturbations imposed by gene deletions. Transcriptome analysis may unravel multiple levels of global gene expression patterns associated with down-regulated transcriptional regulator protein levels complementing insight from regulatory gene knockouts. The carbon metabolism regulator SugR binds to its target promoter DNA sequences with a wide range of affinities in vitro [25,82,83,84,85]. The differences observed in vivo between CRISPRi repression and deletion of the gene *sugR* with regard to decaprenoxanthin accumulation (Figure 8 and Figure 9) support this notion. This may call for an in-depth analysis of the effects of gradually reduced SugR protein levels between wild-type levels and zero in the deletion mutant using CRISPRi combined with genome-wide transcriptome analysis. These analogous vs. digital (CRISPRi vs. deletion) perturbation experiments may also provide insight into the gradual changes at the proteome, metabolome, or fluxome levels [86].

Systems metabolic engineering finely balances enzymes of the pathway of interest (including contributory pathways) using different promoters, ribosome-binding sites, or synthetic operon structures [2,87,88,89] in order to achieve high productivities. Recently, CRISPRi was used to adjust different levels of the arginine biosynthesis repressor ArgR in *E. coli*, which accelerated growth twofold as compared to the deletion of *argR*, while specific arginine production remained similar [90]. In a similar study, CRISPRi was applied to adjust cell growth by repression of key growth-related genes. Upon proper choice of sgRNAs, addition time, and induction level, carbon flux was precisely redistributed between biomass formation and synthesis of *N*-acetyl-glucosamine as the target product, which was produced up to about 90 g L^−1^ [91]. The approach described here may in the future be used for multiplexing since it is likely that CRISPRi multiplex screening will identify synergistic effects if two or more genes are repressed simultaneously by CRISPRi.

**Figure 10:**
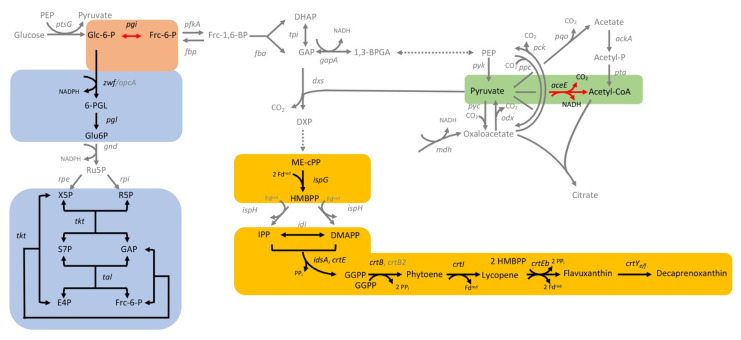
Scheme of *C. glutamicum* metabolism with CRISPRi target genes that significantly improved (red arrows) or reduced (black arrows) decaprenoxanthin biosynthesis in *C. glutamicum* when repressed. Grey is used to depict all other reactions in glycolysis (orange shading), the pentose phosphate pathway (blue shading), the TCA cycle (green shading), as well as the MEP pathway and the carotenogenesis (yellow shading). Abbreviations are explained in Figure 3.

Decaprenoxanthin biosynthesis was significantly improved when *pgi* was repressed by CRISPRi or was deleted (Figure 7, Figure 9 and Figure 10). NADPH in *C. glutamicum* mainly derives from the pentose phosphate pathway [92] and is known to limit l-lysine production [93], especially during growth on carbon sources that support low PPP fluxes, such as acetate [20] or fructose [94,95]. Deletion of *pgi* is known to be beneficial for l-lysine production [96]. While production of 1 molecule of l-lysine requires 4 molecules of NADPH, 35 molecules of reduction equivalents (NADPH, reduced ferredoxin) are required per molecule of decaprenoxanthin. For the biosynthesis of the 10 C5 isoprenoid pyrophosphate precursors of decaprenoxanthin (2 DMAPP, 6 IPP, and 2 HMBPP) from GAP and pyruvate, 38 reduction equivalents are needed: 1 NADPH and 3 reduced ferredoxins per IPP or DMAPP (in the reactions of Dxr, IspG, and IspH) as well as 1 NADPH and 2 reduced ferredoxins per HMBPP (in the reactions of Dxr and IspG). Conversion of the 10 C5 precursors to the C50 decaprenoxanthin yields 3 reduced ferredoxins (1 in the reaction catalyzed by lycopene synthase CrtI and 2 in the elongation reactions catalyzed by lycopene elongase CrtEb; Figure 3). Thus, biosynthesis of decaprenoxanthin imposes a high demand for reduction equivalents to the cell. Improved NADPH provision may also be reached by overexpression of PPP genes, e.g., the genes coding for glucose-6-phosphate dehydrogenase (Zwf) [45], transketolase (Tkt), and transaldolase (Tal) [44]. Engineering of the PPP and the TCA cycle increased β-carotene production in *E. coli* by 64% [44] and in *S. cerevisiae* by 81.4% [45]. The importance of the PPP for decaprenoxanthin production was confirmed here when targeting the genes *tkt*, *tal*, *zwf*, and *pgl* by CRISPRi significantly decreased cellular decaprenoxanthin content (Figure 7b).

Supply of GAP and pyruvate as precursors for decaprenoxanthin biosynthesis was found to be crucial by the CRISPRi analysis (Figure 10). Repression of *aceE* (encoding subunit E1 of the pyruvate dehydrogenase complex that oxidatively decarboxylates pyruvate to acetyl-CoA) increased decaprenoxanthin. Although not statistically significant, CRISPRi of the *fba* gene coding for fructose-1,6-bisphosphate aldolase decreased decaprenoxanthin formation, while CRISPRi of the genes *gapA* and *tpi* encoding GAP converting glycolytic enzymes positively affected decaprenoxanthin formation (Figure 7). Notably, deletion of *gapA* increased the cellular decaprenoxanthin concentration significantly (Figure 9). However, CRISPRi repression and/or deletion of genes central to glycolysis do not come without side effects. For example, deletion of *gapA* cells showed an increased decaprenoxanthin content, but led to lower biomass concentrations; thus, the decaprenoxanthin titer was not higher (compare Figure 9a with Figure 9b). Moreover, deletion of *aceE* has been shown to abolish growth with glucose alone [60], and a positive effect on decaprenoxanthin production was not observable after growth on a glucose–acetate mixture (Figure 9). The results with *aceE* and *gapA* clearly showed that rerouting a major pathway of the central metabolism toward the production of decaprenoxanthin may critically interfere with growth. For example, the central carbon metabolism provides ATP, and indirectly CTP, which are required for isoprenoid pyrophosphate and carotenoid biosynthesis [15]. Thus, targets identified by CRISPRi library screening may require fine balancing of gene expression/repression to optimize decaprenoxanthin production without impairing growth.

Inherent problems in the interpretation of CRISPRi results when targeting an upstream gene of an operon exist due to polar effects on the downstream co-transcribed genes of that operon [97]. The *crtE*-*mmpL*-*crtB*-*crtI*-*crtY_e_*-*crtY_f_*-*crtEb* operon (Figure 6) consists of seven co-transcribed genes [30,33]. CRISPRi repression of the chosen genes of this operon reduced decaprenoxanthin biosynthesis (Figure 5). Besides gene-specific effects, polar effects have to be considered. CRISPRi targeting of the first and second *crt* operon genes (*crtE* and *mmpl*) significantly reduced mRNA levels of *crtEb*, the last gene in the operon (Figure 6). In particular, reduced decaprenoxanthin upon repression of *crtE* came as a surprise since deletion of *crtE* did not abolish carotenogenesis, while deletion of the *crtE* paralog *idsA* did [75]. Therefore, the finding that CRISPRi repression of *idsA* did not reduce decaprenoxanthin was not anticipated (Figure 5b). Since it has been shown that plasmid-borne overexpression of *crtE* compensates for the deletion of *idsA* [75], it is likely that partial repression of *idsA* by CRISPRi was compensated for by CrtE.

## 5. Conclusions

The *C. glutamicum* CRISPRi library designed in this study was shown to be suitable to score targets for improving decaprenoxanthin production, the application example chosen here. The inherent limitations of this screening approach with regard to the transfer of CRISPRi results to clean gene deletions became obvious as they reflect the change from a gradual analogous approach to a digital yes/no approach. With these limitations in mind, many applications of this CRISPRi library and its future extensions become evident, in particular when combined with systems approaches targeted at gaining a basic physiological understanding or at achieving superior biotechnological performance of *C. glutamicum*.

## Figures and Tables

**Table 1:** Strains and plasmids used in this study.

Strain	Characteristics	Reference
	***Corynebacterium glutamicum* strains**	
Wild type (WT)	Wild-type ATCC 13032	[59]
WT Δ*aceE*	*aceE* (cg2466) deletion mutant of WT	[60]
WT Δ*gapA*	*gapA* (cg1791) deletion mutant of WT	[61]
WT Δ*pgi*	*pgi* (cg0973) deletion mutant of WT	[62]
WT Δ*sdhCAB*	*sdhCAB* (cg0445/0447/0448) deletion mutant of WT	This work
WT Δ*sugR*	*sugR* (cg2115) deletion mutant of WT	[25]
MB001	Prophage-cured, genome-reduced WT	[57]
MB001 Δ*crtR*	*crtR* (cg0725) deletion mutant of MB001	[63]
	***E. coli* strains**	
*E. coli* DH5α	F^-^thi^−1^ *endA*1 *hsdr*17(r-, m-) *supE*44 Δ*lacU*169 (Φ80*lacZ*ΔM15) *recA*1 *gyrA*96	[64]
	**Plasmids**	
pK19*mobsacB*	Mobilizable *E. coli* vector used for the construction of insertion and deletion mutants of *C. glutamicum* (*oriV*, *sacB*, *lacZ*α); Kan^R^	[65]
pK19*mobsacB*-Δ*sdhCAB*	pK19*mobsacB* for the deletion of *sdhCAB* (cg0445/0447/0448); Kan^R^	This work
pRG_dCas9	*E. coli/C. glutamicum* shuttle clustered regularly interspaced short palindromic repeats interference (CRISPRi) vector, anhydrotetracycline (aTc)- and IPTG-inducible; Cm^R^	[56]
piCas	*E. coli/B. methanolicus* shuttle CRISPRi vector, mannitol-inducible; Cm^R^	[66]
pS_dCas9	pRG_dCas9 carrying the dCas9 handle followed by the terminator from *S. pyogenes*; Cm^R^	This work
pS_dCas9_*aceA*	pS_dCas9 plasmid carrying the *aceA* (cg2560) sgRNA	This work
pS_dCas9_*aceB*	pS_dCas9 plasmid carrying the *aceB* (cg2559) sgRNA	This work
pS_dCas9_*aceE*	pS_dCas9 plasmid carrying the *aceE* (cg2466) sgRNA	This work
pS_dCas9_*ackA*	pS_dCas9 plasmid carrying the *ackA* (cg3047) sgRNA	This work
pS_dCas9_*acn*	pS_dCas9 plasmid carrying the *acn* (cg1737) sgRNA	This work
pS_dCas9_*crtB*	pS_dCas9 plasmid carrying the *crtB* (cg0721) sgRNA	This work
pS_dCas9_*crtE*	pS_dCas9 plasmid carrying the *crtE* (cg0723) sgRNA	This work
pS_dCas9_*crtEb*	pS_dCas9 plasmid carrying the *crtEb* (cg0717) sgRNA	This work
pS_dCas9_*crtI*	pS_dCas9 plasmid carrying the *crtI* (cg0720) sgRNA	This work
pS_dCas9_*crtR*	pS_dCas9 plasmid carrying the *crtR* (cg0725) sgRNA	This work
pS_dCas9_*crtX*	pS_dCas9 plasmid carrying the *crtX* (cg0730) sgRNA	This work
pS_dCas9_*crtYe*	pS_dCas9 plasmid carrying the *crtYe* (cg0719) sgRNA	This work
pS_dCas9_*deoC*	pS_dCas9 plasmid carrying the *deoC* (cg0458) sgRNA	This work
pS_dCas9_*dxr*	pS_dCas9 plasmid carrying the *dx*r (cg2208) sgRNA	This work
pS_dCas9_*dxs*	pS_dCas9 plasmid carrying the *dxs* (cg2083) sgRNA	This work
pS_dCas9_*eno*	pS_dCas9 plasmid carrying the *eno* (cg1111) sgRNA	This work
pS_dCas9_*fba*	pS_dCas9 plasmid carrying the *fba* (cg3068) sgRNA	This work
pS_dCas9_*fbp*	pS_dCas9 plasmid carrying the *fbp* (cg1157) sgRNA	This work
pS_dCas9_*fixB*	pS_dCas9 plasmid carrying the *fixB* (cg1386) sgRNA	This work
pS_dCas9_*fum*	pS_dCas9 plasmid carrying the *fum* (cg1145) sgRNA	This work
pS_dCas9_*gapA*	pS_dCas9 plasmid carrying the *gapA* (cg1791) sgRNA	This work
pS_dCas9_*gltA*	pS_dCas9 plasmid carrying the *gltA* (cg0949) sgRNA	This work
pS_dCas9_*glxR*	pS_dCas9 plasmid carrying the *glxR* (cg0350) sgRNA	This work
pS_dCas9_*gnd*	pS_dCas9 plasmid carrying the *gnd* (cg1643) sgRNA	This work
pS_dCas9_*icd*	pS_dCas9 plasmid carrying the *icd* (cg0766) sgRNA	This work
pS_dCas9_*idsA*	pS_dCas9 plasmid carrying the *idsA* (cg2384) sgRNA	This work
pS_dCas9_*IIdD*	pS_dCas9 plasmid carrying the *IIdD* (cg3227) sgRNA	This work
pS_dCas9_*ispE*	pS_dCas9 plasmid carrying the *ispE* (cg1039) sgRNA	This work
pS_dCas9_*ispF*	pS_dCas9 plasmid carrying the *ispF* (cg2944) sgRNA	This work
pS_dCas9_*ispG*	pS_dCas9 plasmid carrying the *ispG* (cg2206) sgRNA	This work
pS_dCas9_*ispH*	pS_dCas9 plasmid carrying the *ispH* (cg1164) sgRNA	This work
pS_dCas9_*ldh*	pS_dCas9 plasmid carrying the *ldh* (cg3219) sgRNA	This work
pS_dCas9_*malE*	pS_dCas9 plasmid carrying the *malE* (cg3335) sgRNA	This work
pS_dCas9_*mdh*	pS_dCas9 plasmid carrying the *mdh* (cg2613) sgRNA	This work
pS_dCas9_*mmpl*	pS_dCas9 plasmid carrying the *mmpl* (cg0722) sgRNA	This work
pS_dCas9_*odhA*	pS_dCas9 plasmid carrying the *odhA* (cg1280) sgRNA	This work
pS_dCas9_*odx*	pS_dCas9 plasmid carrying the *odx* (cg1458) sgRNA	This work
pS_dCas9_*opcA*	pS_dCas9 plasmid carrying the *opcA* (cg1779) sgRNA	This work
pS_dCas9_*pck*	pS_dCas9 plasmid carrying the *pck* (cg3169) sgRNA	This work
pS_dCas9_*pfkA*	pS_dCas9 plasmid carrying the *pfkA* (cg1409) sgRNA	This work
pS_dCas9_*pgi*	pS_dCas9 plasmid carrying the *pgi* (cg0973) sgRNA	This work
pS_dCas9_*pgk*	pS_dCas9 plasmid carrying the *pgk* (cg1790) sgRNA	This work
pS_dCas9_*pgl*	pS_dCas9 plasmid carrying the *pgl* (cg1780) sgRNA	This work
pS_dCas9_*pgm*	pS_dCas9 plasmid carrying the *pgm* (cg2800) sgRNA	This work
pS_dCas9_*ppc*	pS_dCas9 plasmid carrying the *ppc* (cg1787) sgRNA	This work
pS_dCas9_*ppsA*	pS_dCas9 plasmid carrying the *ppsA* (cg0644) sgRNA	This work
pS_dCas9_*pqo*	pS_dCas9 plasmid carrying the *pqo* (cg2891) sgRNA	This work
pS_dCas9_*pta*	pS_dCas9 plasmid carrying the *pta* (cg3048) sgRNA	This work
pS_dCas9_*ptsG*	pS_dCas9 plasmid carrying the *ptsG* (cg1537) sgRNA	This work
pS_dCas9_*pyc*	pS_dCas9 plasmid carrying the *pyc* (cg0791) sgRNA	This work
pS_dCas9_*pyk*	pS_dCas9 plasmid carrying the *pyk* (cg2291) sgRNA	This work
pS_dCas9_*ramB*	pS_dCas9 plasmid carrying the *ramB* (cg0444) sgRNA	This work
pS_dCas9_*rpe*	pS_dCas9 plasmid carrying the *rpe* (cg1801) sgRNA	This work
pS_dCas9_*rpi*	pS_dCas9 plasmid carrying the *rpi* (cg2658) sgRNA	This work
pS_dCas9_*rsdA*	pS_dCas9 plasmid carrying the *rsdA* (cg0697) sgRNA	This work
pS_dCas9_*rshA*	pS_dCas9 plasmid carrying the *rshA* (cg0877) sgRNA	This work
pS_dCas9_*sdhA*	pS_dCas9 plasmid carrying the *sdhA* (cg0446) sgRNA	This work
pS_dCas9_*sdhB*	pS_dCas9 plasmid carrying the *sdhB* (cg0447) sgRNA	This work
pS_dCas9_*sdhCD*	pS_dCas9 plasmid carrying the *sdhCD* (cg0445) sgRNA	This work
pS_dCas9_*sigA*	pS_dCas9 plasmid carrying the *sigA* (cg2092) sgRNA	This work
pS_dCas9_*sigB*	pS_dCas9 plasmid carrying the *sigB* (cg2102) sgRNA	This work
pS_dCas9_*sigC*	pS_dCas9 plasmid carrying the *sigC* (cg0309) sgRNA	This work
pS_dCas9_*sigD*	pS_dCas9 plasmid carrying the *sigD* (cg0696) sgRNA	This work
pS_dCas9_*sigE*	pS_dCas9 plasmid carrying the *sigE* (cg1271) sgRNA	This work
pS_dCas9_*sigH*	pS_dCas9 plasmid carrying the *sigH* (cg0876) sgRNA	This work
pS_dCas9_*sigM*	pS_dCas9 plasmid carrying the *sigM* (cg3420) sgRNA	This work
pS_dCas9_*sucC*	pS_dCas9 plasmid carrying the *sucC* (cg2837) sgRNA	This work
pS_dCas9_*sucD*	pS_dCas9 plasmid carrying the *sucD* (cg2836) sgRNA	This work
pS_dCas9_*sugR*	pS_dCas9 plasmid carrying the *sugR* (cg2115) sgRNA	This work
pS_dCas9_*tal*	pS_dCas9 plasmid carrying the *tal* (cg1776) sgRNA	This work
pS_dCas9_*thiE*	pS_dCas9 plasmid carrying the *thiE* (cg2236) sgRNA	This work
pS_dCas9_*tkt*	pS_dCas9 plasmid carrying the *tkt* (cg1774) sgRNA	This work
pS_dCas9_*tpi*	pS_dCas9 plasmid carrying the *tpi* (cg1789) sgRNA	This work
pS_dCas9_*zwf*	pS_dCas9 plasmid carrying the *zwf* (cg1778) sgRNA	This work

## Data Availability

All data are present in the manuscript and its supplement.

## Supplementary Materials

The following are available online at https://www.mdpi.com/2076-2607/9/4/670/s1: Figure S1: Duration of the inhibitory effect of the CRISPRi targeting *crtR*; Table S1: Oligonucleotides used in this study; Table S2: Annealing oligo primers for construction complementary regions of sgRNA templates; Table S3: Data generated with the CRISPRi library for all targets.
