# Supplementary material for: CRISPRi-Library-Guided Target Identification for Engineering Carotenoid Production by Corynebacterium glutamicum
Source: Microorganisms. 2021 Mar 24;9(4):670. doi: 10.3390/microorganisms9040670 (PMC8064071; doi:10.3390/microorganisms9040670)
Supplement: Supplementary file 1 [file microorganisms-09-00670-s001.pdf]

Supplementary Materials to

CRISPRi-library guided target identification for engineering carotenoid production by *Corynebacterium glutamicum*

Vanessa L. Göttl, Ina Schmitt, Kristina Braun, Petra Peters-Wendisch, Volker F. Wendisch * and Nadja A. Henke

Genetics of Prokaryotes, Faculty of Biology & CeBiTec, Bielefeld University, Germany
* Correspondence: Volker.wendisch@uni-bielefeld.de; Tel.: +49-521-106-5611

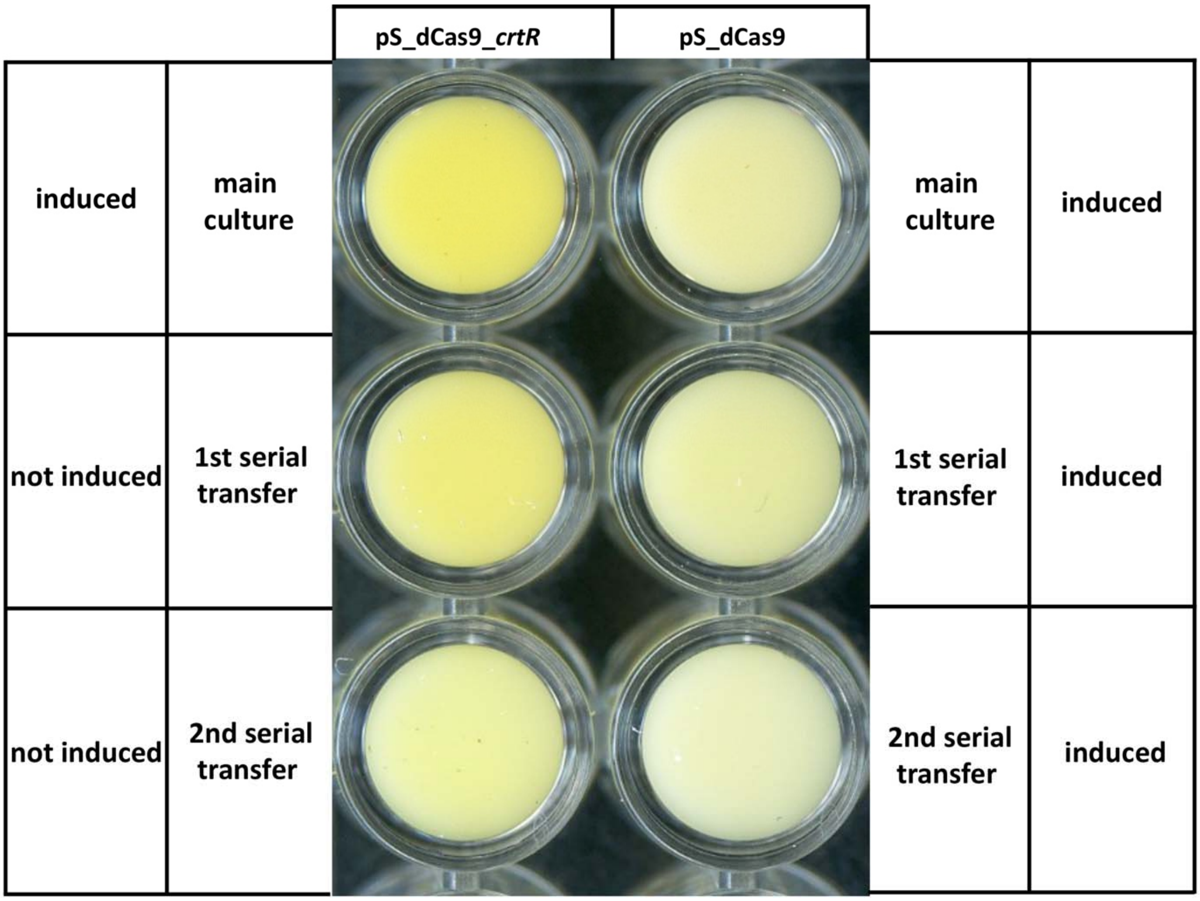

Figure S1. Duration of the inhibitory effect of the CRISPRi targeting *crtR*. For visual comparison of the decaprenoxanthin pigmentation for the duration of the inhibitory effect, the *C. glutamicum* CRISPRi empty vector (pS_dCas9) strain and the strain *C. glutamicum* (pS_dCas9_*crtR*) were induced with 1 mM IPTG and 0.25 µg mL^{-1} aTc and cells were grown in 40 g L^{-1} glucose CGXII minimal medium (main culture induced). Cells were transferred two times into fresh CGXII minimal medium with 40 g L^{-1} glucose (1st and 2nd serial transfer). The empty vector strain was induced under all conditions. The strain *C. glutamicum* (pS_dCas9_*crtR*) was transferred to medium without induction to test the duration of the inhibitory effect. For visual presentation cells were transferred to a 96-well microtiter plate adjusted to an OD_{600} of 150 for comparison. Visible reduction of the decaprenoxanthin level can be observed after two serial transfers of *C. glutamicum* (pS_dCas9_*crtR*) in media without induction of the CRISPRi system.

Table S1. Oligonucleotides used in this study.

Oligonucleotide	Sequence (5′→ 3′)	Used for
vgag	TACGCCAAGCTTGCATGCCTGCAGTTTTAGAGCTAGAAATAGCAAGTTAAAATAAGGCTAGTCCGTTATC	amplification dCas9 handle and terminator from piCas
vgam	TACCCGGGGATCCTCTAGAGTCGACGAAAAAAGCACCGACTCGGTGCC	amplification dCas9 handle and terminator from piCas
vgai	GTATGGCTGTGCAGGTCGTAAA	forward sequencing primer for pS_dCas9
vgaj	CCGCTTCTGCGTTCTGATTTAATCT	reverse sequencing primer for pS_dCas9
vgaw	AGCTCACCTTAAGCTTTCCCC	qRT-PCR *crtR*
vgax	CGGATAACCACTTCACGCTC	qRT-PCR *crtR*
vgjg	TGAGATAGCCGAGGCCCATAAC	qRT-PCR *crtEb*
vgjh	CGCCCCATTAGCTGGATCAATAC	qRT-PCR *crtEb*
vgjc	CCTTGAGCAGTTTCGATGCC	qRT-PCR *idsA*
vgjd	ACCAGCCCACGCATAAAGAG	qRT-PCR *idsA*
vgdr	AACCATCCGTATCCCAGTCC	qRT-PCR *sigA*
Vgdq	AGTCTTCGATGAAGTCGCC	qRT-PCR *sigA*
del-sdhCAB-1	GCTCTAGAGCCACCAGGTGCCCGGGCGT	amplification of flanking region of *sdhCAB*
del-sdhCAB-2	CCCATCCACTAAACTTAAACATCTAACAGTCATGGCACCCTCC	amplification of flanking region of *sdhCAB*
del-sdhCAB-3	TGTTTAAGTTTAGTGGATGGGTTCCGAGGCAAAGACGACTAG	amplification of flanking region of *sdhCAB*
del-sdhCAB-4	GCTCTAGAACTACTGCGTACGGCCGGTT	amplification of flanking region of *sdhCAB*
del-sdhCAB-5	TCCTATAAGTGGGGTTTTATGTCTCTAAACAG	verification of the *sdhCAB* deletion
del-sdhCAB-6	CGCTCGAGGGATGAGTGTGGAGAG	verification of the *sdhCAB* deletion

Table S2: Annealing oligo primers for construction complementary regions of sgRNA templates. The 20 bp target specific regions are given in capital letters.

target	annealing oligo primer sequence (5′→ 3′)
aceA	fw: gccaagcttgcatgcctgcaGCGACGAACGACGCTTGGAA<br>rv: gctatttctagctctaaaacTTCCAAGCGTCGTTCGTCGC
aceB	fw: gccaagcttgcatgcctgcaGAACCAGTTAGAGTAACCTA<br>rv: gctatttctagctctaaaacTAGGTTACTCTAACTGGTTC
aceE	fw: gccaagcttgcatgcctgcaTCTTCACCTGGGTGGTAGTA<br>rv: gctatttctagctctaaaacTACTACCACCCAGGTGAAGA
ackA	fw: gccaagcttgcatgcctgcaGTGTCAAAGACAGCTACGTG<br>rv: gctatttctagctctaaaacCACGTAGCTGTCTTTGACAC
acn	fw: gccaagcttgcatgcctgcaAAGGGTGCTCTTAGCATTGA<br>rv: gctatttctagctctaaaacTCAATGCTAAGAGCACCCTT
crtB	fw: gccaagcttgcatgcctgcaTGGTTTTGGGGAAATAAAAT<br>rv: gctatttctagctctaaaacATTTTATTTCCCCAAAACCA
crtE	fw: gccaagcttgcatgcctgcaTAACATCGTCATGAACCAAA<br>rv: gctatttctagctctaaaacTTTGGTTCATGACGATGTTA
crtEb	fw: gccaagcttgcatgcctgcaCAATCTCTCCTGCATTTAAT<br>rv: gctatttctagctctaaaacATTAAATGCAGGAGAGATTG
crtI	fw: gccaagcttgcatgcctgcaACGTGGAGAAATTATTATAA<br>rv: gctatttctagctctaaaacTTATAATAATTTCTCCACGT
crtX	fw: gccaagcttgcatgcctgcaGGCCAGGCGTCTCCTCTGTA<br>rv: gctatttctagctctaaaacTACAGAGGAGACGCCTGGCC
crtYe	fw: gccaagcttgcatgcctgcaAAAGAATTGCTCGTAACGGA<br>rv: gctatttctagctctaaaacTCCGTTACGAGCAATTCTTT
deoC	fw: gccaagcttgcatgcctgcaAACATCTACTTCGGAAGCTC<br>rv: gctatttctagctctaaaacGAGCTTCCGAAGTAGATGTT
dxr	fw: gccaagcttgcatgcctgcaGAGGCCTTTATTAATAAGGG<br>rv: gctatttctagctctaaaacCCCTTATTAATAAAGGCCT
dxs	fw: gccaagcttgcatgcctgcaGGATCTTATGCACATAGGAC<br>rv: gctatttctagctctaaaacGTCCTATGTGCATAAGATCC
eno	fw: gccaagcttgcatgcctgcaGTTGCGGAGAGCCTCAGAGA<br>rv: gctatttctagctctaaaacTCTCTGAGGCTCTCCGCAAC
fba	fw: gccaagcttgcatgcctgcaGAGGAGGTGCAGTTGATGGC<br>rv: gctatttctagctctaaaacGCCATCAACTGCACCTCCTC
fbp	fw: gccaagcttgcatgcctgcaCCAGCTCCATAGCAAGGTTA<br>rv: gctatttctagctctaaaacTAACCTTGCTATGGAGCTGG
fixB	fw: gccaagcttgcatgcctgcaCGGTACTCAGCTAACACGCC<br>rv: gctatttctagctctaaaacGGCGTGTTAGCTGAGTACCG
fum	fw: gccaagcttgcatgcctgcaTCCAGACCACGACCAGAGAT<br>rv: gctatttctagctctaaaacATCTCTGGTCGTGGTCTGGA
gapA	fw: gccaagcttgcatgcctgcaGTCGCCAGTGTATGCGTGAA<br>rv: gctatttctagctctaaaacTTCACGCATACACTGGCGAC
gltA	fw: gccaagcttgcatgcctgcaAGCCAGTGCTCACATAACCT<br>rv: gctatttctagctctaaaacAGGTTATGTGAGCACTGGCT

target	annealing oligo primer sequence (5′→ 3′)
glxR	fw: gccaagcttgcatgcctgcaTGAGGTTATTGACTGCCGTT<br>rv: gctatttctagctctaaaacAACGGCAGTCAATAACCTCA
gnd	fw: gccaagcttgcatgcctgcaGATTTCCTTCTCGCGACGAA<br>rv: gctatttctagctctaaaacTTCGTCGCGAGAAGGAAATC
icd	fw: gccaagcttgcatgcctgcaCTTGCGGCAGTCTTCGATAA<br>rv: gctatttctagctctaaaacTTATCGAAGACTGCCGCAAG
idsA	fw: gccaagcttgcatgcctgcaCAGCCCACGCATAAAGAGGA<br>rv: gctatttctagctctaaaacTCCTCTTTATGCGTGGGCTG
IIdD	fw: gccaagcttgcatgcctgcaTGCGCGTGTGATTGAGAGTT<br>rv: gctatttctagctctaaaacAACTCTCAATCACACGCGCA
ispE	fw: gccaagcttgcatgcctgcaCGATTCGCACAAGAATACCG<br>rv: gctatttctagctctaaaacCGGTATTCTTGTGCGAATCG
ispF	fw: gccaagcttgcatgcctgcaGCGGCGGGGTCCAAATTTGG<br>rv: gctatttctagctctaaaacCCAAATTTGGACCCCGCCGC
ispG	fw: gccaagcttgcatgcctgcaGTTGCCTGGGTTCACACGAA<br>rv: gctatttctagctctaaaacTTCGTGTGAACCCAGGCAAC
ispH	fw: gccaagcttgcatgcctgcaATTTCTTTACGGACATAAAT<br>rv: gctatttctagctctaaaacATTTATGTCCGTAAAGAAAT
ldh	fw: gccaagcttgcatgcctgcaAGCGGAGTCCAGGACAGTTC<br>rv: gctatttctagctctaaaacGAACTGTCCTGGACTCCGCT
malE	fw: gccaagcttgcatgcctgcaAACACCAGGGGTGTAAGCAA<br>rv: gctatttctagctctaaaacTTGCTTACACCCCTGGTGTT
mdh	fw: gccaagcttgcatgcctgcaTCCAGAAGTTTCAGTTCTAC<br>rv: gctatttctagctctaaaacGTAGAACTGAAACTTCTGGA
mmpl	fw: gccaagcttgcatgcctgcaAAGCCAAACTAAAATTAGCA<br>rv: gctatttctagctctaaaacTGCTAATTTTAGTTTGGCTT
odhA	fw: gccaagcttgcatgcctgcaGGCTGTGCTTCTGTTGTAGC<br>rv: gctatttctagctctaaaacGCTACAACAGAAGCACAGCC
odx	fw: gccaagcttgcatgcctgcaGTGAACTTTGGTTCAGTGAA<br>rv: gctatttctagctctaaaacTTCACTGAACCAAAGTTCAC
opcA	fw: gccaagcttgcatgcctgcaGGTCTTGGAAATTTGCTGGG<br>rv: gctatttctagctctaaaacCCCAGCAAATTTCCAAGACC
pck	fw: gccaagcttgcatgcctgcaTCTTCCTGGCCTGGCTCCAA<br>rv: gctatttctagctctaaaacTTGGAGCCAGGCCAGGAAGA
pfkA	fw: gccaagcttgcatgcctgcaCACACGGTTGTGAGATTCAG<br>rv: gctatttctagctctaaaacCTGAATCTCACAACCGTGTG
pgi	fw: gctatttctagctctaaaacCTGAATCTCACAACCGTGTG<br>rv: gctatttctagctctaaaacCGTTTCTGTGTTGGAAGACC
pgk	fw: gccaagcttgcatgcctgcaGGCAATGATTCGGCCCTTAT<br>rv: gctatttctagctctaaaacATAAGGGCCGAATCATTGCC
pgl	fw: gccaagcttgcatgcctgcaTGGACTCAGAATCACTGACA<br>rv: gctatttctagctctaaaacTGTCAGTGATTCTGAGTCCA
pgm	fw: gccaagcttgcatgcctgcaGCCATCACGAGGAGGGTTGT<br>rv: gctatttctagctctaaaacACAACCCTCCTCGTGATGGC
ppc	fw: gccaagcttgcatgcctgcaTGCGGCGGCGAGTCTCAGTT<br>rv: gctatttctagctctaaaacAACTGAGACTCGCCGCCGCA
ppsA	fw: gccaagcttgcatgcctgcaCCACCGAGTACTTCTAGAAC<br>rv: gctatttctagctctaaaacGTTCTAGAAGTACTCGGTGG

target	annealing oligo primer sequence (5′→ 3′)
pqo	fw: gccaagcttgcatgcctgcaCAATCTGGGCACTCGGAATA<br>rv: gctatttctagctctaaaacTATTCCGAGTGCCCAGATTG
pta	fw: gccaagcttgcatgcctgcaGTCCATCGACGCATAGTTCT<br>rv: gctatttctagctctaaaacAGAACTATGCGTCGATGGAC
ptsG	fw: gccaagcttgcatgcctgcaCAATCCACGAGTACTTGCCA<br>rv: gctatttctagctctaaaacTGGCAAGTACTCGTGGATTG
pyc	fw: gccaagcttgcatgcctgcaCTTCAGAAGCAAAAGAGCGG<br>rv: gctatttctagctctaaaacCCGCTCTTTTGCTTCTGAAG
pyk	fw: gccaagcttgcatgcctgcaCAAGACGGATCTTCGGTCCT<br>rv: gctatttctagctctaaaacAGGACCGAAGATCCGTCTTG
ramB	fw: gccaagcttgcatgcctgcaTCGGTGATGCGCAATAACAC<br>rv: gctatttctagctctaaaacGTGTTATTGCGCATCACCGA
rpe	fw: gccaagcttgcatgcctgcaCCAACACCTGCTCCCCTAAG<br>rv: gctatttctagctctaaaacCTTAGGGGAGCAGGTGTTGG
rpi	fw: gccaagcttgcatgcctgcaCTTCGTGGCCGTGGGCCTTA<br>rv: gctatttctagctctaaaacTAAGGCCCACGGCCACGAAG
rsdA	fw: gccaagcttgcatgcctgcaGGTTGTCACTGTCTGTACCT<br>rv: gctatttctagctctaaaacAGGTACAGACAGTGACAACC
rshA	fw: gccaagcttgcatgcctgcaAATCCGCAGACGCTCGCAGG<br>rv: gctatttctagctctaaaacCCTGCGAGCGTCTGCGGATT
sdhA	fw: gccaagcttgcatgcctgcaGTTCCTTAGGGGACCAGATG<br>rv: gctatttctagctctaaaacCATCTGGTCCCCTAAGGAAC
sdhB	fw: gccaagcttgcatgcctgcaACGTGGAGGGTATCAGCGTC<br>rv: gctatttctagctctaaaacGACGCTGATACCCTCCACGT
sdhCD	fw: gccaagcttgcatgcctgcaCGACCAGCAGGATAATACGT<br>rv: gctatttctagctctaaaacACGTATTATCCTGCTGGTCG
sigA	fw: gccaagcttgcatgcctgcaGACTCAGATGCGACTCCCAA<br>rv: gctatttctagctctaaaacTTGGGAGTCGCATCTGAGTC
sigB	fw: gccaagcttgcatgcctgcaTCATGACTTCACGCTCTATC<br>rv: gctatttctagctctaaaacGATAGAGCGTGAAGTCATGA
sigC	fw: gccaagcttgcatgcctgcaCCGGGCTAGCGATAGTAGCC<br>rv: gctatttctagctctaaaacGGCTACTATCGCTAGCCCGG
sigD	fw: gccaagcttgcatgcctgcaGCGAGCATAACGCAAAACAA<br>rv: gctatttctagctctaaaacTTGTTTTGCGTTATGCTCGC
sigE	fw: gccaagcttgcatgcctgcaAGACACGCATGAATGTTTCT<br>rv: gctatttctagctctaaaacAGAAACATTCATGCGTGTCT
sigH	fw: gccaagcttgcatgcctgcaCTTTCGGTACATGTTGATGT<br>rv: gctatttctagctctaaaacACATCAACATGTACCGAAAG
sigM	fw: gccaagcttgcatgcctgcaGAGCTGCTTCTGCTCTATAA<br>rv: gctatttctagctctaaaacTTATAGAGCAGAAGCAGCTC
sucC	fw: gccaagcttgcatgcctgcaGAGCCGACGTCGGTGCCACA<br>rv: gctatttctagctctaaaacTGTGGCACCGACGTCGGCTC
sucD	fw: gccaagcttgcatgcctgcaTCGCCGGGAGTAATAATGCC<br>rv: gctatttctagctctaaaacGGCATTATTACTCCCGGCGA
sugR	fw: gccaagcttgcatgcctgcaTTGGCCAGATTAAGTGCGAT<br>rv: gctatttctagctctaaaacATCGCACTTAATCTGGCCAA
tal	fw: gccaagcttgcatgcctgcaTGCCGGAAGTAATGCGCTCG<br>rv: gctatttctagctctaaaacCGAGCGCATTACTTCCGGCA

target	annealing oligo primer sequence (5′→ 3′)
thiE	fw: gccaagcttgcatgcctgcaCGGGAGTCCTTCGCGCATTA<br>rv: gctatttctagctctaaaacTAATGCGCGAAGGACTCCCG
tkt	fw: gccaagcttgcatgcctgcaTCTCATCTGCATCCCATGTT<br>rv: gctatttctagctctaaaacAACATGGGATGCAGATGAGA
tpi	fw: gccaagcttgcatgcctgcaGCCCTCAACGAGAGTCTGGA<br>rv: gctatttctagctctaaaacTCCAGACTCTCGTTGAGGGC
zwf	fw: gccaagcttgcatgcctgcaGCGGTTTGCTAGATCATAAA<br>rv: gctatttctagctctaaaacTTTATGATCTAGCAAACCGC

Table S3: Data generated with the CRISPRi library for all targets. ΔOD, growth rate and decaprenoxanthin production are listed. Mean values of two biological duplicates are given.

target	ΔOD_{600}	growth rate [h^{-1}]	decaprenoxanthin [mg (g CDW)$^{-1}$]
aceA	43.6 ± 2.0	0.33 ± 0.00	0.09 ± 0.00
aceB	39.6 ± 4.2	0.34 ± 0.02	0.08 ± 0.01
aceE	30.9 ± 1.1	0.18 ± 0.02	0.11 ± 0.01
ackA	45.9 ± 0.3	0.30 ± 0.01	0.08 ± 0.00
acn	48.1 ± 1.3	0.16 ± 0.00	0.05 ± 0.00
crtB	45.3 ± 0.9	0.34 ± 0.00	0.01 ± 0.00
crtE	40.5 ± 1.7	0.32 ± 0.01	0.02 ± 0.00
crtEb	47.5 ± 0.3	0.32 ± 0.00	0.01 ± 0.00
crtI	46.0 ± 0.8	0.30 ± 0.00	0.00 ± 0.00
crtR	43.0 ± 5.6	0.17 ± 0.01	0.44 ± 0.05
crtX	38.0 ± 2.4	0.30 ± 0.01	0.07 ± 0.00
crtYe	34.9 ± 5.9	0.32 ± 0.01	0.02 ± 0.00
deoC	38.7 ± 1.7	0.27 ± 0.03	0.03 ± 0.00
dxr	41.0 ± 0.2	0.25 ± 0.02	0.04 ± 0.00
dxs	40.2 ± 2.6	0.31 ± 0.01	0.02 ± 0.01
eno	45.8 ± 0.6	0.20 ± 0.00	0.06 ± 0.00
fba	42.6 ± 1.6	0.25 ± 0.01	0.04 ± 0.00
fbp	58.4 ± 3.4	0.30 ± 0.00	0.05 ± 0.00
fixB	30.5 ± 1.5	0.14 ± 0.00	0.02 ± 0.00
fum	41.7 ± 3.3	0.33 ± 0.01	0.07 ± 0.01
gapA	36.4 ± 1.0	0.24 ± 0.01	0.07 ± 0.00
gltA	56.1 ± 1.5	0.28 ± 0.01	0.05 ± 0.00
glxR	41.9 ± 3.7	0.33 ± 0.00	0.15 ± 0.01
gnd	38.4 ± 0.6	0.20 ± 0.01	0.04 ± 0.00
icd	44.7 ± 0.7	0.23 ± 0.00	0.05 ± 0.01
idsA	44.5 ± 0.9	0.30 ± 0.01	0.06 ± 0.01
IIdD	58.5 ± 3.1	0.33 ± 0.01	0.05 ± 0.00
ispE	43.1 ± 3.5	0.34 ± 0.01	0.06 ± 0.01
ispF	45.5 ± 3.3	0.31 ± 0.00	0.07 ± 0.01
ispG	43.7 ± 3.5	0.35 ± 0.00	0.01 ± 0.00
ispH	33.4 ± 5.2	0.17 ± 0.02	0.03 ± 0.00
ldh	36.3 ± 3.1	0.29 ± 0.01	0.06 ± 0.01
malE	39.3 ± 0.3	0.25 ± 0.01	0.08 ± 0.00
mdh	43.5 ± 1.1	0.34 ± 0.00	0.06 ± 0.00
mmpl	43.2 ± 1.8	0.32 ± 0.01	0.01 ± 0.00
odhA	44.4 ± 1.6	0.24 ± 0.01	0.08 ± 0.02
odx	57.9 ± 0.1	0.29 ± 0.00	0.05 ± 0.00
opcA	43.6 ± 1.2	0.29 ± 0.01	0.04 ± 0.01
pck	55.1 ± 1.5	0.31 ± 0.02	0.06 ± 0.00
pfkA	57.6 ± 0.2	0.21 ± 0.00	0.06 ± 0.00
pgi	55.1 ± 3.7	0.28 ± 0.01	0.10 ± 0.00
pgk	37.8 ± 0.4	0.26 ± 0.01	0.06 ± 0.00
pgl	39.6 ± 0.4	0.20 ± 0.01	0.02 ± 0.00
pgm	55.3 ± 4.5	0.31 ± 0.00	0.05 ± 0.01
ppc	46.0 ± 1.2	0.27 ± 0.01	0.04 ± 0.00
ppsA	55.7 ± 0.3	0.31 ± 0.01	0.05 ± 0.00
pqo	55.8 ± 2.6	0.31 ± 0.01	0.06 ± 0.00

target	ΔOD_{600}	growth rate [h^{-1}]	decaprenoxanthin [mg (g CDW)$^{-1}$]
pta	45.5 ± 0.7	0.30 ± 0.00	0.08 ± 0.00
ptsG	52.9 ± 1.1	0.21 ± 0.00	0.06 ± 0.00
pyc	54.8 ± 2.4	0.31 ± 0.00	0.06 ± 0.01
pyk	52.8 ± 1.6	0.38 ± 0.01	0.05 ± 0.01
ramB	27.5 ± 0.1	0.19 ± 0.04	0.07 ± 0.04
rpe	45.6 ± 1.2	0.27 ± 0.00	0.07 ± 0.02
rpi	30.2 ± 0.8	0.26 ± 0.06	0.04 ± 0.02
rsdA	38.8 ± 0.4	0.30 ± 0.00	0.07 ± 0.00
rshA	46.2 ± 1.0	0.31 ± 0.00	0.04 ± 0.01
sdhA	45.0 ± 1.0	0.34 ± 0.01	0.04 ± 0.00
sdhB	45.9 ± 2.5	0.32 ± 0.00	0.04 ± 0.00
sdhCD	52.8 ± 3.2	0.32 ± 0.01	0.03 ± 0.00
sigA	8.5 ± 2.7	0.06 ± 0.01	0.08 ± 0.01
sigB	44.1 ± 0.3	0.28 ± 0.01	0.09 ± 0.00
sigC	45.3 ± 1.7	0.31 ± 0.00	0.06 ± 0.00
sigD	35.5 ± 4.3	0.32 ± 0.01	0.08 ± 0.01
sigE	42.9 ± 0.1	0.31 ± 0.01	0.06 ± 0.00
sigH	44.1 ± 0.9	0.31 ± 0.00	0.06 ± 0.00
sigM	41.7 ± 0.7	0.32 ± 0.02	0.06 ± 0.00
sucC	46.5 ± 0.3	0.31 ± 0.01	0.07 ± 0.00
sucD	47.9 ± 0.5	0.34 ± 0.01	0.07 ± 0.00
sugR	28.3 ± 3.9	0.15 ± 0.00	0.03 ± 0.01
tal	36.6 ± 0.6	0.24 ± 0.05	0.02 ± 0.00
thiE	35.4 ± 4.0	0.19 ± 0.01	0.06 ± 0.01
tkt	37.2 ± 2.4	0.19 ± 0.00	0.02 ± 0.00
tpi	40.5 ± 1.1	0.32 ± 0.00	0.07 ± 0.00
zwf	38.1 ± 0.7	0.19 ± 0.00	0.02 ± 0.00
